# An international Delphi consensus for surgical quality assessment of lymphadenectomy and anastomosis in minimally invasive total gastrectomy for gastric cancer

**DOI:** 10.1007/s00464-023-10614-9

**Published:** 2023-12-26

**Authors:** Amila Cizmic, Ivan Romic, Andrea Balla, Nicolò Barabino, Gabriele Anania, Gian Luca Baiocchi, Branko Bakula, Carmen Balagué, Felix Berlth, Vasile Bintintan, Umberto Bracale, Jan-Hendrik Egberts, Hans F. Fuchs, Suzanne S. Gisbertz, Ines Gockel, Peter Grimminger, Richard van Hillegersberg, Noriyuki Inaki, Arul Immanuel, Daniel Korr, Philipp Lingohr, Pietro Mascagni, Nathaniel Melling, Marco Milone, Yoav Mintz, Salvador Morales-Conde, Yusef Moulla, Beat P. Müller-Stich, Kiyokazu Nakajima, Magnus Nilsson, Matthias Reeh, Pierpaolo Sileri, Eduardo M. Targarona, Yuki Ushimaru, Young-Woo Kim, Sheraz Markar, Felix Nickel, Anuja T. Mitra

**Affiliations:** 1 Department of General, Visceral and Thoracic Surgery, University Medical Center Hamburg-Eppendorf, Martinistraße 52, 20246 Hamburg, Germany; 2 Department of Hepatobiliary Surgery & Liver Transplantation, University Hospital Centre Zagreb, Zagreb, Croatia; 3 Coloproctology and Inflammatory Bowel Disease Surgery Unit, IRCCS San Raffaele Scientific Institute, Milan, Italy; 4 Department of Surgical Sciences & Integrated Diagnostic, University of Genoa, Genoa, Italy; 5 Department of Medical Science, University of Ferrara, 4121 Ferrara, Italy; 6 Department of Clinical and Experimental Sciences, University of Brescia, Brescia, Italy; 7 Department of Surgery, University Hospital Sveti Duh, Zagreb, Croatia; 8 Department of General and Digestive Surgery, Hospital de la Santa Creu I Sant Pau, Autonomous University of Barcelona, Barcelona, Spain; 9 Department of General, Visceral and Transplant Surgery, University Medical Center of the Johannes Gutenberg University, Mainz, Germany; 10 Department of Surgery, University Hospital Cluj Napoca, Cluj-Napoca, Romania; 11 General and Emergency Surgical Unit, Department of Medicine, Surgery and Dentistry, University of Salerno, AOU San Giovanni and Ruggi D’Aragona, Salerno, Italy; 12 Department of Surgery, Israelit Hospital, Hamburg, Germany; 13 Department of General, Visceral, Cancer and Transplantation Surgery, University Hospital Cologne, Cologne, Germany; 14 Department of Surgery, Amsterdam UMC Location, University of Amsterdam, Amsterdam, The Netherlands; 15 Cancer Treatment and Quality of Life, Cancer Center Amsterdam, Amsterdam, The Netherlands; 16 Department of Visceral, Transplant, Thoracic and Vascular Surgery, University Hospital of Leipzig, Leipzig, Germany; 17 Department of Surgery, University Medical Center Utrecht, Utrecht University, Utrecht, The Netherlands; 18 Department of Gastrointestinal Surgery/Breast Surgery, Kanazawa University Hospital, Kanazawa, Ishikawa Japan; 19 Northern Oesophago-Gastric Unit, Newcastle Upon Tyne Hospitals NHS Trust, Newcastle Upon Tyne, UK; 20 Department for General, Visceral, Thoracic and Vascular Surgery, University Hospital of Bonn, Bonn, Germany; 21 Fondazione Policlinico Universitario Agostino Gemelli IRCCS, Rome, Italy; 22 Institute of Image-Guided Surgery, IHU-Strasbourg, Strasbourg, France; 23 Department of Clinical Medicine and Surgery, University of Naples “Federico II”, 80131 Naples, Italy; 24 Department of General Surgery, Hadassah Hebrew University Medical Center, Jerusalem, Israel; 25 Department of General and Digestive Surgery, University Hospital Virgen Macarena, School of Medicine of the University of Seville, Seville, Spain; 26 Unit of General and Digestive Surgery, Hospital Quironsalud Sagrado Corazon, Seville, Spain; 27 Department of Digestive Surgery, University Digestive Healthcare Center Basel, Basel, Switzerland; 28 Department of Next Generation Endoscopic Intervention, Department of Gastroenterological Surgery, Graduate School of Medicine, Osaka University, Osaka, Japan; 29 Department of Clinical Science, Intervention and Technology, Karolinska Institute, Karolinska University Hospital, Stockholm, Sweden; 30 Department of General, Visceral and Vascular Surgery, Marienkrankenhaus, Hamburg, Germany; 31 Coloproctology and Inflammatory Bowel Disease Surgery Unit, IRCCS San Raffaele Scientific Institute, Vita-Salute University, Milan, Italy; 32 Surgery Unit, Hospital de la Santa Creu i Sant Pau, Barcelona, Spain; 33 Department of Gastroenterological Surgery, Osaka University Graduate School of Medicine, Osaka, Japan; 34 Center for Gastric Cancer, National Cancer Center, Goyang, Gyeonggi Republic of Korea; 35 Nuffield Department of Surgical Sciences, University of Oxford, Oxford, UK; 36 Department of Surgery & Cancer, Imperial College London, London, UK

**Keywords:** Minimally invasive surgery, Laparoscopy, Gastrectomy, Lymphadenectomy, Delphi, International, Technical skills, Consensus, Surgical quality assessment

## Abstract

**Background:**

Minimally invasive total gastrectomy (MITG) is a mainstay for curative treatment of patients with gastric cancer. To define and standardize optimal surgical techniques and further improve clinical outcomes through the enhanced MITG surgical quality, there must be consensus on the key technical steps of lymphadenectomy and anastomosis creation, which is currently lacking. This study aimed to determine an expert consensus from an international panel regarding the technical aspects of the performance of MITG for oncological indications using the Delphi method.

**Methods:**

A 100-point scoping survey was created based on the deconstruction of MITG into its key technical steps through local and international expert opinion and literature evidence. An international expert panel comprising upper gastrointestinal and general surgeons participated in multiple rounds of a Delphi consensus. The panelists voted on the issues concerning importance, difficulty, or agreement using an online questionnaire. A priori consensus standard was set at > 80% for agreement to a statement. Internal consistency and reliability were evaluated using Cronbach's *α*.

**Results:**

Thirty expert upper gastrointestinal and general surgeons participated in three online Delphi rounds, generating a final consensus of 41 statements regarding MITG for gastric cancer. The consensus was gained from 22, 12, and 7 questions from Delphi rounds 1, 2, and 3, which were rephrased into the 41 statetments respectively. For lymphadenectomy and aspects of anastomosis creation, Cronbach’s *α* for round 1 was 0.896 and 0.886, and for round 2 was 0.848 and 0.779, regarding difficulty or importance.

**Conclusions:**

The Delphi consensus defined 41 steps as crucial for performing a high-quality MITG for oncological indications based on the standards of an international panel. The results of this consensus provide a platform for creating and validating surgical quality assessment tools designed to improve clinical outcomes and standardize surgical quality in MITG.

**Supplementary Information:**

The online version contains supplementary material available at 10.1007/s00464-023-10614-9.

Gastric cancer accounts for Europe's fourth highest cause of cancer-related deaths, and its incidence is projected to rise globally [1–3]. The mainstay of curative treatment is surgical resection with corresponding en-bloc D2 lymphadenectomy (LND) [4, 5]. International adoption of minimally invasive techniques in treating gastric cancer has been observed [6–11]. First performed by Kitano [12], it has subsequently been evaluated in several large-scale randomized controlled trials (RCTs) in the Asian continent [13–16] and corroborated in high-quality European population-based studies and RCTs [17, 18]. Outcomes from these studies have advocated the use of minimally invasive total gastrectomy (MITG) by demonstrating superiority in short- and long-term general surgical outcomes such as post-operative length of stay [4, 15, 19] and equivalence of MITG for oncological specific outcomes. Multicenter studies such as the LOGICA and the STOMACH trials demonstrated the feasibility and oncological efficacy of MITG compared to open surgery and subsequently recommended this approach to improve long-term outcomes [17, 20–22].

For oncological indications, key technical steps specific to gastric cancer must be respected, such as the extent and the number of lymph nodes harvested and achieving an R0 resection margin. Therefore, MITG for oncological treatment is considered an advanced minimally invasive procedure as it involves achieving complete en-bloc dissection of lymph node stations and creating a high-quality anastomosis [23, 24]. The lack of compliance with the D2 LND in MITG has been reported in several publications and can negatively impact patient outcomes [18, 25, 26]. Combined with the technical challenges, there is heterogeneity within techniques and steps surgeons undertake to achieve a complete oncological MITG. These variations can impact clinical outcomes in patients, complicate reporting and comparing outcomes between studies, hinder the assessment of surgical quality, and pose challenges to the teaching and training of MITG.

The aim of this study was to develop an international expert consensus on the technical steps of LND and general aspects of anastomosis creation in MITG for oncological indications using the Delphi method. The consensus will inform the development of surgical quality assessment tools for MITG and the creation of a technical framework to aid skill acquisition and surgeon training. The consensus will also enable benchmarking of this procedure so practicing surgeons and certifying bodies are ensured that MITG is being performed competently and, most importantly, safely [27].

## Materials and methods

**Fig. 1:**
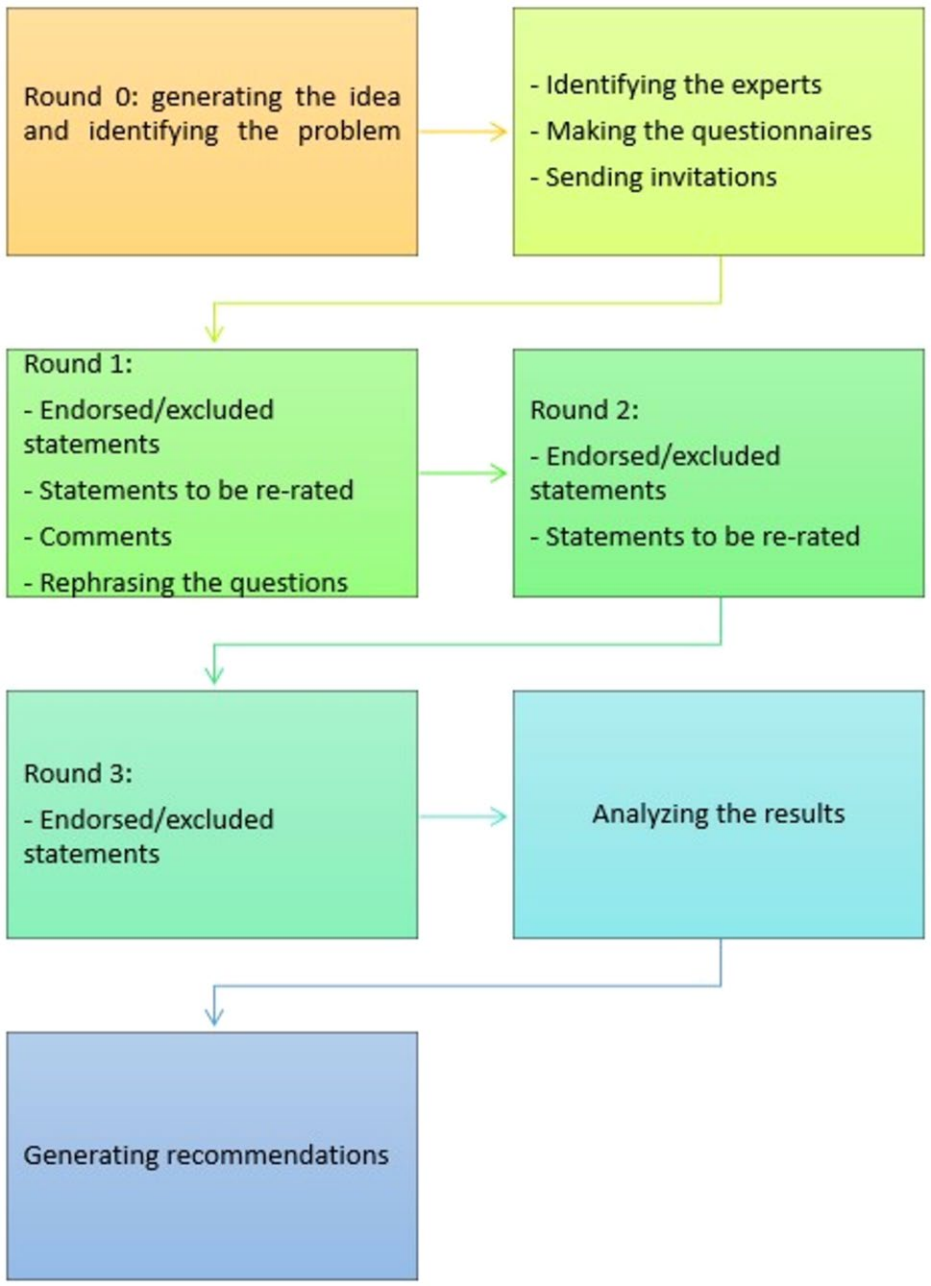
Flowchart of the study

This study utilized the Delphi method to achieve consensus by soliciting international expert opinion on essential technical steps in LND and general aspects of anastomosis creation required to perform MITG for oncological indications (Fig. 1). The study utilized the AGREE II framework for the assessment of the methodological quality of practice guidelines (Appendices 1 and 2). AGREE II comprises 23 items organized into six quality domains: (i) scope and purpose; (ii) stakeholder involvement; (iii) rigor of development; (iv) clarity of presentation; (v) applicability; and (vi) editorial independence that targets various aspects of practice guideline quality [28]. The outcomes of this study were to create consensus on the most important steps of the operation required to achieve optimal oncological outcomes, minimize technical heterogeneity, and inform the training of future surgeons.

### The Delphi method

The Delphi method was first developed by the RAND Corporation in 1948. It is a structured and iterative process used to acquire knowledge and opinions from experts on a selected topic [29, 30]. The major advantages of using the Delphi method are that a wide range of views can be obtained, which enables the collation of international opinions while being cost-effective and eliminating the need to travel. It also provides a platform for experts to revise their initial opinions based on the feedback from the group, fostering convergence and consensus-building. The anonymous nature of the Delphi method also ensures that a single dominant group member does not inordinately influence the group's outcome [31]. The Delphi method has been widely used in determining guidelines and recommendations in the medical field, especially on topics that lack RCTs as evidence [27, 32–34].

### Creation of MITG scoping questionnaire

A novel 100-step scoping questionnaire was created by deconstructing the entire MITG procedure into key technical steps focusing on D2 LND and general aspects of anastomosis creation for oncological indications (see Appendices 3, 4, and 5 for the complete questionnaire). The deconstruction of procedures into their individual elements has been described as a validated and effective strategy [35]. The MITG sub-steps were created based on author group consensus, expert supervision from upper gastrointestinal (UGI) surgical faculty at the University Medical Center Hamburg-Eppendorf, Germany, and the University of Oxford, UK, and peer-reviewed published literature. The survey was refined through iterations. The final version consisted of questions related to the demographics of the expert panel, and the remaining questions were categorized into two main groups: questions surrounding LND and general aspects associated with the creation of the anastomosis.

### Selection of experts

Expert surgeons in UGI were identified globally to represent an international consensus on performing MITG in Asian and European countries. Experts were selected based on the following inclusion criteria: high case volume defined as > 20 cases per year in their local institution, national or international board accreditations, and individual credentialing of surgeons based on their number of index cases performed as well as specific expertise in methodology. All panelists were identified as experts in their clinical field and currently practicing minimally invasive surgeons. General surgeons were also considered for the expert panel since some countries do not differentiate between subspecialties despite having UGI high case volume centers. In these cases, the determining inclusion factors were the high case volume center and the UGI expertise of the panelists. Thirty participants representing 12 countries were initially approached through e-mail with personalized invitation letters explaining the project. The membership of the expert panel was anonymized.

### Delphi rounds

Statements generated in the scoping questionnaire were presented to the expert panel via an online survey on SurveyMonkey® (USA). The questions were divided into three domains depending on the type of possible answers: questions with a 5-point Likert scale regarding the importance of the subject, questions with a 5-point Likert scale regarding the difficulty of the subject, and binary ‘yes’ or ‘no’ questions. An a priori consensus standard was set at > 80% if the panel deemed the statement important and < 40% unimportant. Panelists were also encouraged to suggest additional statements or modifications to the statements in free text fields. Rounds 2 and 3 questions were modified according to the experts' comments and suggestions from previous rounds. Each round was closed once 90% of the experts had responded. Results were analyzed, and those questions that failed to reach consensus were evaluated, rephrased if necessary, and distributed to the same experts for rounds 2 and 3 of the survey, in addition to novel statements generated from the previous rounds. The survey was closed when all responses reached consensus or agreed on non-consensus.

### Statistical analysis

Statistical analysis and descriptive statistics were performed using the SPSS software (version 25.0, IBM SPSS Inc., Chicago, Illinois, USA), and data were given as absolute frequency and mean ± standard deviation. Cronbach's *α* was calculated to determine internal consistency.

## Results

Thirty UGI/general surgery experts were invited to participate in the Delphi survey. Twenty-eight (93.3%) experts across 12 countries completed the first, and 25 (83.3%) completed the second and third Delphi round. The demographic characteristics of the experts are presented in Table 1.

**Table 1:** Demographic characteristics of the Delphi participants (*n* = 30)

Country of residence *n* (%)	
Germany	10 (33.3)
Italy	6 (20.0)
Japan	2 (6.7)
Spain	3 (10.0)
Netherlands	2 (6.7)
Croatia	1 (3.3)
Israel	1 (3.3)
Romania	1 (3.3)
Sweden	1 (3.3)
Switzerland	1 (3.3)
United Kingdom	1 (3.3)
South Korea	1 (3.3)
Specialty	
UGI surgery	17 (56.7)
General surgery	13 (43.3)
Years of experience in MIS	
3–5 years	2 (6.7)
6–10 years	1 (3.3)
11–15 years	6 (20.0)
16–20 years	6 (20.0)
> 20 years	15 (50.0)

*n* number, *UGI* upper gastrointestinal, *MIS* minimally invasive surgery

Seventeen out of 30 experts were UGI surgeons (56.7%), and 13 (43.3%) were general surgeons. The majority of experts had more than 20 years experience in MIS.

The total time needed to complete all three rounds was seven months (11/2022–06/2023). In all three groups, there were 100 questions, 65 with 5-Likert scale answers regarding the importance, 19 with 5-Likert scale answers regarding the difficulty, and 16 with yes or no answers (Table 2).

**Table 2:** Characteristics of the Delphi rounds

Parameters	Round 1	Round 2	Round 3
Number of participants	28	25	25
The time needed to close the round (months)	4	2	1
Average time spent to finish the survey (min)	19.4	13.4	2.4
Questions in total	69	34	12
Demographic questions	11 (15.9)	2 (6.3)	2 (16.7)
Questions based on difficulty *n* (%)	12 (17.4)	7 (21.9)	0 (0.0)
Cronbach’s *α* for questions based on difficulty	0.896	0.848	–
Questions based on importance *n* (%)	46 (66.7)	19 (59.4)	0 (0.0)
Cronbach’s *α* for questions based on importance	0.886	0.779	–
Questions with y/n answers	0 (0.0)	6 (18.8)	10 (83.3)
Consensus reached *n* (%)	22 (37.9)	12 (37.5)	7 (70)
Questions that did not reach consensus *n* (%)	36 (62.1)	20 (62.5)	3 (30)
Questions moved to the next round* *n* (%)	26 (44.8)	9 (28.1)	0 (0.0)**
Questions excluded *n* (%)	10 (17.2)	11 (34.4)	0 (0.0)**

*n* number, *y/n* yes or no

*Some questions were rephrased into two or more questions

**Last Delphi round

The Cronbach's *α* for round 1 questions regarding importance (*n* = 46, 66.7%) was 0.886. For questions regarding the difficulty (*n* = 12, 17.4%) in round 1, Cronbach's *α* was 0.896. In round 2, Cronbach's *α* was 0.779 for questions regarding importance (*n* = 19, 59.4%) and 0.848 for questions regarding difficulty (*n* = 7, 21.9%) (Table 2).

Consensus was reached from 22 questions from round 1, 12 from round 2, and 7 from round 3 (Table 2). These were rephrased into 41 consensus statements regarding LND and general aspects of anastomosis creation in MITG (Table 3). The 41 statements were divided into general aspects of the MITG (*n* = 2), key technical aspects of LND (*n* = 33), general aspects of LND in MITG (*n* = 1), and general aspects of anastomosis creation (*n* = 5) (Table 3).

**Table 3:** Consensus statements regarding LND and general aspects of the anastomosis creation in MITG

Statements	Cons. (%)
General aspects of the MITG	
The division of the gastrocolic ligament is extremely/very important to facilitate LND	96
Omentectomy can be omitted in MITG in T1/2 tumor stages	84
Station 3	
LND station 3 is extremely/very important	86
LND station 3 at the lesser gastric curvature, reaching cardia and the right crux of the diaphragm, is extremely/very important	89
Station 4d	
LND station 4d is extremely/very important in MITG	96
Identifying the gastroduodenal artery in LND station 4d is extremely/very important	82
In LND station 4d, it is extremely/very important to follow the gastroduodenal artery to identify the right gastroepiploic artery	82
Performing the LND station 4d along the distal right gastroepiploic artery is extremely/very important	96
LND station 4d: It is extremely/very important to ligate the right gastroepiploic artery at its origin	93
It is extremely/very important to identify and ligate the right gastroepiploic vein and/or the gastrocolic trunk to facilitate the LND of stations 4d and 6	82
Station 11p	
LND station 11p is extremely/very important in MITG	86
Identifying the proximal splenic artery in LND station 11p is extremely/very important	93
Station 4sb	
LND station 4sb is extremely/very important in MITG	82
The identification of the left gastroepiploic vessels is an extremely/very important step toward station 4sb LND	96
The ligation of the left gastroepiploic artery and vein close to their origin is important in LND station 4sb	84
Station 4sa	
LND 4sa is extremely/very important in tumor localization on greater gastric curvature, fundus, or corpus of the stomach	88
In LND station 4sa, it is extremely/very important to identify and ligate the short gastric vessels	88
In LND station 4sa, it is extremely/very important to continue the dissection until the angle of His and the left crus of the diaphragm	86
Station 7	
LND station 7 is extremely/very important in MITG	100
In LND 7, it is extremely/very important to retract the vascular pedicle of the stomach upwards to improve visualization and dissect the LGA and LGV	92
In LND station 7, it is extremely/very important to ligate the LGA at its origin at the celiac trunk	93
In LND station 7, it is extremely/very important to ligate the LGV at the upper border of the pancreas	84
Station 9	
LND station 9 is extremely/very important in MITG	86
Stations 5 and 6	
LND of stations 5 and 6 is extremely/very important in MITG	86
In the LND of stations 5 and 6, it is extremely/very important to dissect the posterior side of the postpyloric part of the duodenum to create a safe passage for the stapler	96
In the LND of stations 5 and 6, it is extremely/very important to perform a stapled division of the duodenum 1–2 cm postpyloric	89
Stations 8a and 12a	
LND stations 8a and 12a are extremely/very important in MITG	82
In the LND of stations 8a and 12a, the identification of the common hepatic artery is extremely/very important	86
In the LND of stations 8a and 12a, the identification of the proper hepatic artery by following the superior border of the common hepatic artery is extremely/very important	86
In LND of stations 8a and 12a, the ligation of the RGA at its origin is extremely/very important	89
Stations 1 and 2	
LND of stations 1 and 2 is extremely/very important in MITG	82
It is extremely/very important to dissect the right (station 1) and left (station 2) paracardial lymph nodes along the right and left crux of the diaphragm	80
Station 10	
LND station 10 is extremely/very important for complete oncological dissection in tumors of greater gastric curvature at the level of the spleen	96
LND station 10 can be omitted for tumors far from the greater gastric curvature and the spleen	92
LND of the splenic hilum (station 10) is very difficult/difficult in MITG	86
General aspects of LND in MITG	
It is extremely/very important that the lymph nodes are intact	82
General aspects of anastomosis in MITG	
Skillful creation of the anastomosis is extremely/very important in MITG	100
Safety and efficiency in creating the anastomosis in MITG are extremely/very important	100
Tension-free and torsion-free anastomosis in MITG is extremely/very important	93
The frozen section should be performed if the tumor localization is perceived to be close to the margin	92
An intraoperative leak test can be performed at the surgeon's discretion to rule out the leakage	80

*LND* lymphadenectomy, *Cons. (%)* percentage of answers that reached consensus, *MITG* minimally invasive total gastrectomy, *LGA* left gastric artery, *LGV* left gastric vein, *RGA* right gastric artery

### Statements of non-consensus

A certain number of questions were excluded from rounds 1 and 2 due to either incorporation into the other questions or irrelevance (round 1 *n* = 10, round 2 *n* = 11).

Eight questions with a 5-point Likert scale on difficulty performing LND of stations 4sa, 5, 6, 7, 1, 2, 9, 8a, 12a, and 11d required rephrasing due to heterogeneity of answers after rounds 1 and 2 (Table 4). Five questions with a 5-point Likert scale regarding the importance of performing certain MITG steps did not reach an agreement after 3 Delphi rounds, and three questions regarding general aspects of the anastomosis creation with binary responses did not reach consensus (Table 4).

**Table 4:** Areas of less agreement

Questions regarding importance	EI (%)	I (%)	S (%)	NSI (%)	NI (%)
How important is the omentectomy, irrespective of the tumor stage?	4	8	24	56	8
How important is the omentectomy for advanced tumor stages (T3/T4)?	8	28	36	20	8
How important is identifying the superior mesenteric vein as a necessary step of LND for MITG?	0	12	24	48	16
How important is the station 4sa LND, irrespective of tumor localization?	0	36	52	12	0
Station 11d LND: how important is the division of the posterior gastric vessels at their origin from the splenic artery?	4	52	36	8	0

Questions regarding difficulty	E (%)	V/E (%)	N (%)	D (%)	V/D (%)
How difficult is the LND along the SGV (station 4sa)?	3	32	50	11	4
How difficult is the LND along the inferior and superior border of the duodenum (stations 5 and 6)?	0	18	43	36	3
How difficult is the LND station 7 along the LGA and LGV?	6	20	32	38	4
How difficult are the LNDs of stations 1 and 2?	18	43	32	7	0
How difficult is the LND along the celiac trunk (station 9)?	3	8	39	39	11
How difficult is it to perform a sufficient LND along the common hepatic artery (station 8a)?	0	28	32	28	12
How difficult is it to perform a sufficient LND along the proper hepatic artery (station 12a)?	0	12	28	48	12
How difficult is the LND along the distal splenic artery (station 11d) without splenectomy?	0	4	44	44	8

Questions with y/n answers	Yes (%)			No (%)	
Should the frozen section always be performed if proximal margin resection is less than 6 cm and/or in case of doubt about tumor-free margins?	60			40	
Following neoadjuvant therapy, is an R0 resection oncologically acceptable irrespective of the resection distance from the margin?	68			32	
Should an assessment of the perfusion of the anastomosis be performed with fluorescence or hyperspectral imaging at the discretion of the surgeon?	60			40	

*EI* extremely important, *I* important, *S* somewhat important, *NSI* not so important, *N* not at all important, *E* easy, *VE* very easy, *N* neither easy nor difficult, *D* difficult, *VD* very difficult, *MITG* minimally invasive total gastrectomy, *LND* lymphadenectomy, *SGV* short gastric vessels, *LGA* left gastric artery, *LGV* left gastric vein, *y/n* yes or no

### AGREE II checklist

Two independent assessors evaluated the methodological quality of the guidelines using the AGREE II checklist. Twenty-three items divided into six domains were evaluated based on the practice guidelines (Appendix 1). All domains had a score > 70% defined previously as a quality threshold (domain 1: 92%, domain 2: 78%, domain 3: 84%, domain 4: 92%, domain 5: 75% and domain 6: 96%) (Appendix 2).

## Discussion

This is the first international Delphi consensus on the recommended technical steps regarding LND and general aspects of anastomosis creation in MITG for oncological indications. This study aimed to establish and summarize the current recommendations on technical steps required to achieve a high-quality MITG for gastric cancer, explicitly focusing on LND and general anastomosis creation. Thirty international UGI and general surgery experts from 12 countries participated in this study, answering 100 questions through 3 Delphi rounds, generating 41 final recommended statements. The study met the quality threshold of > 70% for all six domains of the AGREE II checklist [28].

The majority of the final consensus statements were attained in round 1 of the Delphi, with high interrater reliability and overall homogeneity in their MITG operating technique. This indicates there was general alignment among how experts perform MITG, validating the selection of our expert panel.

The results of this study identified several issues regarding the importance and perceived difficulty of performing LND in MITG. Statements that commonly reached consensus related to the importance of how to technically approach of the D2 LND steps. The areas of least consensus related to the perceived difficulty of performing LND of specific lymph node stations. For example, 12% of experts rated performing a sufficient LND along the proper hepatic artery (station 12a) as easy, whereas 12% reported it as very difficult. A potential reason for heterogeneous responses on LND difficulty could be the different experience levels of panelists not only with gastrectomy but also with other index procedures that involve similar operative steps or not, such as bariatric surgeries, hiatal hernia repairs, esophagectomies, or pancreatic surgeries, which might influence the difficulty perception of performing specific surgical steps in MITG. However, no difference in the difficulty statement was detected regarding the volume and experience of the panelists. The only ‘difficulty’ statement where the panelists reached agreement was that station 10 LND is extremely/very difficult, which correlates to MITG's challenges regarding the LND of the splenic hilum [36].

Other than defining aspects related to LND in MITG, it was important to determine the general aspects of the procedure, such as omentectomy and division of the gastrocolic ligament. Interestingly, the panelists agreed that performing omentectomy can be omitted in early tumor stages (T1/T2). However, the necessity of omentectomy in advanced stages (T3/T4) or irrespective of tumor stage did not reach consensus after three rounds despite having been recommended in many guidelines and available literature [37–40].

The final domain focused on general aspects of anastomosis creation in MITG. Due to the various technical approaches to performing the esophagojejunostomy in MITG, the statements focused on common facets of anastomosis creation rather than choosing one specific way of performing it. The panelists agreed that skillful, safe, efficient, tension- and torsion-free anastomosis is extremely or very important in MITG. The recommendation from round 3 that a frozen section should be performed if the tumor is perceived to be close to the resection margin also reached consensus (92%). However, the panelists could not agree on whether a frozen section should always be performed if the proximal resection margin was less than 6 cm (60% yes vs. 40% no) in round 2. Some surgical steps regarding anastomosis were still heterogenous among panelists, such as whether the R0 resection is oncologically acceptable irrespective of the resection margin distance after neoadjuvant therapy (68% yes vs. 32% no) and regarding the assessment of the perfusion of anastomosis. The panelists were asked whether the assessment of the perfusion of the anastomosis should be performed with indocyanine green fluorescence or a hyperspectral imaging camera, which revealed that 60% of experts stated that it should be performed and 40% of the panel reported it can be omitted. This result may reflect the relatively new use of such perfusion assessment tools in MITG, so the answers may change in the future to reflect the routine incorporation of these tools [41] in case scientific evidence can prove definitive advantages. Regarding the need for anastomosis assessment using an intraoperative leakage test, the panelists reached a consensus that it could be performed at the discretion of the operating surgeon to rule out leakage. This may reflect differences in clinical practice and the absence of evidence in favor of or against routine leak tests. This may also be a statistical problem since intraoperative detection of leaks represents a rather rare finding and thus may be difficult to handle statistically. On the other hand, leak tests may be of clinical value for detecting technical problems while not posing significant risks or added operative time or cost.

The strengths of this study are the solicitation of global expert opinion unifying views across Asia and Europe and the quality and experience of the experts taking part in this consensus. One of the advantages is the high expert response rate (93%) and low attrition rates (10%) between rounds and the high interrater agreement demonstrated by Cronbach's *α*, strengthening the content validity and effectiveness of the Delphi method. Furthermore, this study addressed a clinically important issue regarding MITG. Determining a sufficient level of agreement with performing an oncologically acceptable MITG is essential for several reasons. MITG is a complex surgical procedure that requires specialized knowledge and skills. The emphasis on compliance in D2 LND has been proven to be of great importance to patient outcomes [18, 25]. Obtaining a consensus from experts in the field ensures that the recommendations and guidelines for performing the procedure are based on the collective wisdom and experience of skilled surgeons. This helps establish a standard of care and minimizes the variability in surgical techniques, leading to standardized patient outcomes. The Delphi method also drives best practices in medicine since consensus-driven guidelines facilitate knowledge of the most effective and evidence-based approaches, leading to improved patient care. This also helps identify potential risks and develop strategies to mitigate them, thereby enhancing patient safety. By standardizing the procedure and promoting adherence to established guidelines, the quality of care provided to patients undergoing MITG can be improved, reducing the likelihood of adverse events and improving surgical safety.

Such consensus-based guidelines can be incorporated into training programs and surgical curricula to ensure that surgeons receive appropriate education and mentoring in MITG, fostering the development of competent and skilled practitioners. Most importantly, this research is essential for innovation. Delphi consensus can guide the direction of future studies, enabling the focus to shift toward the advancement of MITG. Additionally, consensus guidelines may promote innovation by encouraging the evaluation of new techniques, technologies, and approaches through well-designed research studies.

General limitations of the Delphi method and this study should be recognized. Although the Delphi method should be performed with anonymized panelists to mitigate potential bias and enhance quality and objectivity, this study required the identification of the panelists to ensure the participation of the uttermost experts in MITG. The Delphi method may also be susceptible to the ‘groupthink’ phenomena, where participants tend to conform to a dominant viewpoint or consensus rather than providing independent and diverse opinions. One way of mitigating this effect was the conscious decision to conduct the study online. However, this approach did hinder the establishment of meaningful discussions and clarifying responses on site, as panelists could not directly interact or engage in a real-time dialogue with each other or the researchers. Despite this Delphi consensus being created using a scoping strategy with international input, not all aspects of MITG have been addressed, for example, the optimal limb length and the preferences of exact anastomosis technique (e.g., handsewn versus circular stapler versus linear stapler technique) for anastomosis creation. Another limitation of this study is the lack of investigation about technical aspects or approaches; however, these were not the primary aim of the study, and they will be the object of further studies since the current study focused on oncological outcome and perioperative outcome with regards to general surgical technique.

The results of this study represent the first phase of the ANALYTIQs study that resulted from international collaboration in the EAES Research Sandpit in June 2022. The next step is creating a surgical quality assessment (SQA) tool and its application in video assessments of MITG. This will serve SQA in prospective and randomized studies to ensure optimized surgical performance [42]. The results of the applied SQA will also be used to develop artificial intelligence-based algorithms for automation of SQA. The long-term aims are to determine an SQA of this technique through video-based learning to ensure standardization and improve patient outcomes after MITG for oncological indications. Furthermore, the SQA tool could be used as an intraoperative guide for residents or young surgeons, with the final aim to shorten the learning curve and improve intra- and postoperative results. The predefined surgical steps that emphasize the most important aspects of LND of each lymph node station in MITG for gastric cancer could provide valuable structure in training young surgeons to master this procedure and ensure the best possible patient outcomes through technical impeccability.

In conclusion, this is the first study to determine the crucial technical steps in LND in MITG, deconstructing the integral elements within a complex minimally invasive procedure. It has identified the key technical steps of LND and general aspects of anastomosis creation required to perform adequate MITG for oncological indications by soliciting international expert opinion through a three-round Delphi consensus method. These statements should serve the standardization of the MITG and, through that, the improvement of oncological outcomes through adequate LND.

### Supplementary Information

Below is the link to the electronic supplementary material.

Supplementary file1 (XLSX 12 kb)

Supplementary file2 (PDF 173 kb)

Supplementary file3 (PDF 25 kb)

Supplementary file4 (PDF 127 kb)

Supplementary file5 (PDF 69 kb)
