# Supplementary material for: An international Delphi consensus for surgical quality assessment of lymphadenectomy and anastomosis in minimally invasive total gastrectomy for gastric cancer
Source: Surg Endosc. 2023 Dec 26;38(2):488–98. doi: 10.1007/s00464-023-10614-9 (PMC10830761; doi:10.1007/s00464-023-10614-9)
Supplement: Supplementary file 3 — Supplementary file3 (PDF 25 kb) [file 464_2023_10614_MOESM3_ESM.pdf]

Description of the survey

This is the third round of the Delphi Questionnaire for the Quality Assessment of Anastomosis and Lymphadenectomy in Laparoscopic Total Gastrectomy (The ANALYTIQS Study).

D2 Lymphadenectomy

* 1. Name

* 2. Institution

* 3. Is the division of the gastrocolic ligament an important step of total gastrectomy and to facilitate lymphadenectomy?

- Yes
- No

* 4. Is the identification of the left gastroepiploic vessels an important step toward station 4sb lymphadenectomy?

- Yes
- No

* 5. Is the ligation of the left gastroepiploic artery and vein close to their origin important in station 4sb lymphadenectomy?

- Yes
- No

* 6. Is the station 10 lymphadenectomy important for complete oncological dissection in patients with tumors of greater curvature at the level of the spleen?

- Yes
- No

* 7. Can station 10 lymphadenectomy be omitted for tumors that are far away from the greater curvature of the stomach and the spleen?

- Yes
- No

* 8. Can omentectomy be omitted for T1/T2 tumor stages?

- ◯ Yes
- ◯ No

* 9. Should the frozen section always be performed if proximal margin resection is less than 6 cm and/or in case of doubt about tumor-free margins?

- ◯ Yes
- ◯ No

* 10. Following neoadjuvant therapy, is an R0 resection oncologically acceptable irrespective of the resection distance from the margin?

- ◯ Yes
- ◯ No

* 11. General aspects of the anastomosis: Should an assessment of the perfusion of the anastomosis be performed with fluorescence or hyperspectral imaging at the discretion of the surgeon?

- ◯ Yes
- ◯ No

* 12. An intraoperative leak test may be performed at the discretion of the operating surgeon to rule out anastomotic leaks?

- ◯ Yes
- ◯ No
