# Supplementary material for: An international Delphi consensus for surgical quality assessment of lymphadenectomy and anastomosis in minimally invasive total gastrectomy for gastric cancer
Source: Surg Endosc. 2023 Dec 26;38(2):488–98. doi: 10.1007/s00464-023-10614-9 (PMC10830761; doi:10.1007/s00464-023-10614-9)
Supplement: Supplementary file 4 — Supplementary file4 (PDF 127 kb) [file 464_2023_10614_MOESM4_ESM.pdf]

Description of the survey

The first ten questions are related to the current position and experience in the surgical field.
The survey regarding the procedure itself starts with question 11.

Type of answers (from the Q 11)

Difficulty = level of experience and skill required to complete the task effectively:

(1) Easy (requires little experience and no use of alternative strategies to accomplish regularly)

(3) Medium (requires some experience and use of alternative strategies to accomplish regularly)

(5) Difficult (requires significant experience and use of alternative strategies to accomplish regularly)

Importance = how important is this task for accomplishing good outcomes of the surgery.

(1) Not important/Optional (patient outcomes unlikely to be affected by this task)

(3) Important (patient outcomes likely to be directly affected by this task)

(5) Absolutely important (patient outcomes likely to be directly affected by this task)

* 1. Name

* 2. Institution

* 3. What is your current position?

- ◯ Surgeon in training
- ◯ Attending surgeon
- ◯ Senior attending surgeon

Comments/Suggestions

* 4. What is your specialty?

- ◯ General surgery
- ◯ Upper GI surgery
- ◯ HPB surgery
- ◯ Colorectal surgery

Other (please specify)

* 5. How many years of experience do you have in minimally invasive surgery?

- <2
- 3-5
- 6-10
- 11-15
- 16-20
- >20

* 6. How many laparoscopic total gastrectomies have you performed as a leading surgeon in total?

- <10
- 11-20
- 21-30
- 31-40
- >40

* 7. How many laparoscopic total gastrectomies do you perform per year as a leading surgeon?

- <15
- 16-25
- 26-35
- 36-45
- 46-55
- 56-60
- >60

* 8. What proportion of laparoscopic total gastrectomies do you perform compared to open gastrectomies?

* 9. How many open total gastrectomies have you performed as a leading surgeon in total?

- ◯ <20
- ◯ 21-30
- ◯ 31-40
- ◯ 41-50
- ◯ 51-60
- ◯ >60

* 10. How many open total gastrectomies do you perform as a leading surgeon per year?

- ◯ <10
- ◯ 11-20
- ◯ 21-30
- ◯ 31-40
- ◯ 41-50
- ◯ >50

* 11. How many laparoscopic total gastrectomies are required to complete a learning curve for the procedure?

- ◯ <10
- ◯ 11-20
- ◯ 21-30
- ◯ 31-40
- ◯ >40

Other (please specify)

* 12. Opening of the lesser omentum

- ◯ Extremely important
- ◯ Very important
- ◯ Somewhat important
- ◯ Not so important
- ◯ Not at all important

Comments/Suggestions

* 13. Lymphadenectomy group 3

- Extremely important
- Very important
- Somewhat important
- Not so important
- Not at all important

Comments/Suggestions

* 14. Lymphadenectomy group 3: Dissection at the lesser curvature, reaching cardia and the right crux of the diaphragm

- Extremely important
- Very important
- Somewhat important
- Not so important
- Not at all important

Comments/Suggestions

* 15. Lymphadenectomy group 3: Dissection of the gastrocolic ligament

- Extremely important
- Very important
- Somewhat important
- Not so important
- Not at all important

Comments/Suggestions

* 16. Lymphadenectomy group 3: Dissection towards the middle colic vessels and the inferior border of the pancreatic body and tail

- Extremely important
- Very important
- Somewhat important
- Not so important
- Not at all important

Comments/Suggestions

* 17. Lymphadenectomy group 3: Identification of the superior mesenteric vein

- Extremely important
- Very important
- Somewhat important
- Not so important
- Not at all important

Comments/Suggestions

* 18. Lymphadenectomy group 3: Identification of the gastrocolic trunk

- Extremely important
- Very important
- Somewhat important
- Not so important
- Not at all important

Comments/Suggestions

* 19. Lymphadenectomy group 4d

- Extremely important
- Very important
- Somewhat important
- Not so important
- Not at all important

Comments/Suggestions

* 20. Lymphadenectomy group 4d: Identification of the gastroduodenal artery

- Extremely important
- Very important
- Somewhat important
- Not so important
- Not at all important

Comments/Suggestions

* 21. Lymphadenectomy group 4d: Follow the gastroduodenal artery to identify the right gastroepiploic artery

- ◯ Extremely important
- ◯ Very important
- ◯ Somewhat important
- ◯ Not so important
- ◯ Not at all important

Comments/Suggestions

* 22. Lymphadenectomy group 4d: Lymph node dissection along the right gastroepiploic artery

- ◯ Extremely important
- ◯ Very important
- ◯ Somewhat important
- ◯ Not so important
- ◯ Not at all important

Comments/Suggestions

* 23. Lymphadenectomy group 4d: Ligate the right gastroepiploic artery at its origin

- ◯ Extremely important
- ◯ Very important
- ◯ Somewhat important
- ◯ Not so important
- ◯ Not at all important

Comments/Suggestions

* 24. Lymphadenectomy group 4d: Identification and ligation of the right gastroepiploic vein at the junction with the gastrocolic trunk

- ◯ Extremely important
- ◯ Very important
- ◯ Somewhat important
- ◯ Not so important
- ◯ Not at all important

Comments/Suggestions

* 25. Lymphadenectomy group 11p

- Extremely important
- Very important
- Somewhat important
- Not so important
- Not at all important

Comments/Suggestions

* 26. Lymphadenectomy group 11p: Identify the proximal splenic artery

- Extremely important
- Very important
- Somewhat important
- Not so important
- Not at all important

Comments/Suggestions

* 27. Lymphadenectomy group 11p: Lymph node dissection along the proximal splenic artery

- Very easy
- Easy
- Neither easy nor difficult
- Difficult
- Very difficult

Comments/Suggestions

* 28. Lymphadenectomy group 11p: Identify the left gastroepiploic artery and vein

- Extremely important
- Very important
- Somewhat important
- Not so important
- Not at all important

Comments/Suggestions

* 29. Lymphadenectomy group 4sb

- Extremely important
- Very important
- Somewhat important
- Not so important
- Not at all important

Comments/Suggestions

* 30. Lymphadenectomy group 4sb: Lymph node dissection along the left gastroepiploic artery and vein

- Very easy
- Easy
- Neither easy nor difficult
- Difficult
- Very difficult

Comments/Suggestions

* 31. Lymphadenectomy group 4sb: Ligate the left gastroepiploic artery and vein at least below the bifurcation of the first gastric branch

- Extremely important
- Very important
- Somewhat important
- Not so important
- Not at all important

Comments/Suggestions

* 32. Lymphadenectomy group 4sa

- Extremely important
- Very important
- Somewhat important
- Not so important
- Not at all important

Comments/Suggestions

* 33. Lymphadenectomy group 4sa: Identify the short gastric vessels

- Extremely important
- Very important
- Somewhat important
- Not so important
- Not at all important

Comments/Suggestions

* 34. Lymphadenectomy group 4sa: Lymph node dissection along the short gastric vessels

- Very easy
- Easy
- Neither easy nor difficult
- Difficult
- Very difficult

Comments/Suggestions

* 35. Lymphadenectomy group 4sa: Ligate the short gastric vessels

- Extremely important
- Very important
- Somewhat important
- Not so important
- Not at all important

Comments/Suggestions

* 36. Lymphadenectomy group 4sa: Continue dissection until the angle of His and the left crus

- Extremely important
- Very important
- Somewhat important
- Not so important
- Not at all important

Comments/Suggestions

* 37. Lymphadenectomy group 7

- Extremely important
- Very important
- Somewhat important
- Not so important
- Not at all important

Comments/Suggestions

* 38. Lymphadenectomy group 7: Place a retractor underneath the stomach and retract it upwards

- Extremely important
- Very important
- Somewhat important
- Not so important
- Not at all important

Comments/Suggestions

* 39. Lymphadenectomy group 7: Lymph node dissection along the left gastric artery and vein

- Very easy
- Easy
- Neither easy nor difficult
- Difficult
- Very difficult

Comments/Suggestions

* 40. Lymphadenectomy group 7: Division of the left gastric artery at its origin at the celiac trunk

- Extremely important
- Very important
- Somewhat important
- Not so important
- Not at all important

Comments/Suggestions

* 41. Lymphadenectomy group 7: Division of the left gastric vein at the portal vein

- Extremely important
- Very important
- Somewhat important
- Not so important
- Not at all important

Comments/Suggestions

* 42. Lymphadenectomy group 9

- Extremely important
- Very important
- Somewhat important
- Not so important
- Not at all important
- Comments/Suggestions

* 43. Lymphadenectomy group 9: Lymph node dissection along the celiac trunk

- Very easy
- Easy
- Neither easy nor difficult
- Difficult
- Very difficult

Comments/Suggestions

* 44. Lymphadenectomy groups 5 and 6

- Extremely important
- Very important
- Somewhat important
- Not so important
- Not at all important

Comments/Suggestions

* 45. Lymphadenectomy groups 5 and 6: Dissection of the posterior side of the postpyloric part of the duodenum to create a safe passage of the stapler

- Extremely important
- Very important
- Somewhat important
- Not so important
- Not at all important

Comments/Suggestions

* 46. Lymphadenectomy groups 5 and 6: Lymph node dissection along the inferior and superior border of the duodenum

- Very easy
- Easy
- Neither easy nor difficult
- Difficult
- Very difficult

Comments/Suggestions

* 47. Lymphadenectomy groups 5 and 6: Division of the duodenum with 1-2 cm postpyloric

- Extremely important
- Very important
- Somewhat important
- Not so important
- Not at all important

Comments/Suggestions

* 48. Lymphadenectomy groups 8a, 8p, and 12a

- Extremely important
- Very important
- Somewhat important
- Not so important
- Not at all important

Comments/Suggestions

* 49. Lymphadenectomy groups 8a, 8p, and 12a: Opening the gastroduodenal ligament at the level of the hepatoduodenal ligament

- Extremely important
- Very important
- Somewhat important
- Not so important
- Not at all important

Comments/Suggestions

* 50. Lymphadenectomy groups 8a, 8p, and 12a: Identifying the common hepatic artery

- Extremely important
- Very important
- Somewhat important
- Not so important
- Not at all important

Comments/Suggestions

* 51. Lymph node dissection along the common hepatic artery (group 8a)

- Very easy
- Easy
- Neither easy nor difficult
- Difficult
- Very difficult

Comments/Suggestions

* 52. Lymph node dissection of the porta, and common hepatic artery from above, down, and below (group 8p)

- Very easy
- Easy
- Neither easy nor difficult
- Difficult
- Very difficult

Comments/Suggestions

* 53. Lymphadenectomy groups 8a, 8p, and 12a: Following the superior border of the common hepatic artery, identification of the proper hepatic artery

- Extremely important
- Very important
- Somewhat important
- Not so important
- Not at all important

Comments/Suggestions

* 54. Lymph node dissection along the proper hepatic artery (group 12a)

- Very easy
- Easy
- Neither easy nor difficult
- Difficult
- Very difficult

Comments/Suggestions

* 55. Lymphadenectomy groups 8a, 8p, and 12a: ligation of right gastric artery

- Extremely important
- Very important
- Somewhat important
- Not so important
- Not at all important

Comments/Suggestions

* 56. Lymphadenectomy groups 1 and 2

- Extremely important
- Very important
- Somewhat important
- Not so important
- Not at all important

Comments/Suggestions

* 57. Dissection of the right (group 1) and left (group 2) paracardial lymph nodes along the right and left crus

- Very easy
- Easy
- Neither easy nor difficult
- Difficult
- Very difficult

Comments/Suggestions

* 58. Lymphadenectomy groups 11d and 10

- Extremely important
- Very important
- Somewhat important
- Not so important
- Not at all important

Comments/Suggestions

* 59. Lymphadenectomy groups 11d and 10: Division of the posterior gastric vessels at their origin from the splenic artery

- Extremely important
- Very important
- Somewhat important
- Not so important
- Not at all important

Comments/Suggestions

* 60. Lymph node dissection along the distal splenic artery (group 11d)

- Very easy
- Easy
- Neither easy nor difficult
- Difficult
- Very difficult

Comments/Suggestions

* 61. Lymph node dissection of the splenic hilum (group 10)

- Very easy
- Easy
- Neither easy nor difficult
- Difficult
- Very difficult

Comments/Suggestions

* 62. Are resected lymph nodes intact?

- Extremely important
- Very important
- Somewhat important
- Not so important
- Not at all important

Comments/Suggestions

* 63. Performing omentectomy

- Extremely important
- Very important
- Somewhat important
- Not so important
- Not at all important

Comments/Suggestions

* 64. General aspects of the anastomosis: Transection at least 5 cm proximally to the tumor

- Extremely important
- Very important
- Somewhat important
- Not so important
- Not at all important

Comments/Suggestions

* 65. General aspects of the anastomosis: Skillful creation of the anastomosis

- Extremely important
- Very important
- Somewhat important
- Not so important
- Not at all important

Comments/Suggestions

* 66. General aspects of the anastomosis: Safety and efficiency

- Extremely important
- Very important
- Somewhat important
- Not so important
- Not at all important

Comments/Suggestions

* 67. General aspects of the anastomosis: Assessment of the vascularization (subjective visual, hyperspectral camera imaging, ICG, or other)

- Extremely important
- Very important
- Somewhat important
- Not so important
- Not at all important

Comments/Suggestions

* 68. General aspects of the anastomosis: Leakage test

- Extremely important
- Very important
- Somewhat important
- Not so important
- Not at all important

Comments/Suggestions

* 69. General aspects of the anastomosis: Tension- and torsion-free anastomosis

- ◯ Extremely important
- ◯ Very important
- ◯ Somewhat important
- ◯ Not so important
- ◯ Not at all important

Comments/Suggestions

[ ]
