# Supplementary material for: An international Delphi consensus for surgical quality assessment of lymphadenectomy and anastomosis in minimally invasive total gastrectomy for gastric cancer
Source: Surg Endosc. 2023 Dec 26;38(2):488–98. doi: 10.1007/s00464-023-10614-9 (PMC10830761; doi:10.1007/s00464-023-10614-9)
Supplement: Supplementary file 5 — Supplementary file5 (PDF 69 kb) [file 464_2023_10614_MOESM5_ESM.pdf]

Description of the survey

This is the second round of the Delphi Questionnaire for the Quality Assessment of Anastomosis and Lymphadenectomy in Laparoscopic Total Gastrectomy (The ANALYTIQS Study).

Type of answers

Difficulty = level of experience and skill required to complete the task effectively:

(1) Easy (requires little experience and no use of alternative strategies to accomplish regularly)

(3) Medium (requires some experience and use of alternative strategies to accomplish regularly)

(5) Difficult (requires significant experience and use of alternative strategies to accomplish regularly)

Importance = how important is this task for accomplishing good outcomes of the surgery?

(1) Not important/Optional (patient outcomes unlikely to be affected by this task)

(3) Important (patient outcomes likely to be directly affected by this task)

(5) Absolutely important (patient outcomes likely to be directly affected by this task)

D2 Lymphadenectomy

* 1. Name

* 2. Institution

* 3. Is the dissection of the gastrocolic ligament an important step of total gastrectomy and to facilitate lymphadenectomy?

- Extremely important
- Very important
- Somewhat important
- Not so important
- Not at all important

Comments/Suggestions

* 4. How important is identifying the superior mesenteric vein as a necessary step of lymphadenectomy for total gastrectomy?

- Extremely important
- Very important
- Somewhat important
- Not so important
- Not at all important

Comments/Suggestions

* 5. Station 4d lymphadenectomy: how important is the identification and ligation of the right gastroepiploic vein at its root or ligation of the gastrocolic trunk?

- Extremely important
- Very important
- Somewhat important
- Not so important
- Not at all important

Comments/Suggestions

* 6. How important is the identification of the left gastroepiploic artery and vein as a step towards station 4sb lymphadenectomy?

- Extremely important
- Very important
- Somewhat important
- Not so important
- Not at all important

Comments/Suggestions

* 7. How important is the station 4sa lymphadenectomy in case of tumor localization on greater curvature, fundus, or corpus of the stomach?

- Extremely important
- Very important
- Somewhat important
- Not so important
- Not at all important

Comments/Suggestions

* 8. How important is the station 4sa lymphadenectomy, irrespective of tumor localization?

- Extremely important
- Very important
- Somewhat important
- Not so important
- Not at all important

Comments/Suggestions

* 9. Station 4sa lymphadenectomy: how important is it to identify and ligate the short gastric vessels?

- Extremely important
- Very important
- Somewhat important
- Not so important
- Not at all important

Comments/Suggestions

* 10. Station 4sa lymphadenectomy: how important is it to ligate the left gastroepiploic artery and vein close to their origin?

- Extremely important
- Very important
- Somewhat important
- Not so important
- Not at all important

Comments/Suggestions

* 11. How difficult is the station 4sa lymphadenectomy?

- Very easy
- Easy
- Neither easy nor difficult
- Difficult
- Very difficult

Comments/Suggestions

* 12. How important is it to retract the vascular pedicle of the stomach upwards to improve visualization and dissect the left gastric artery and vein in station 7 lymphadenectomy?

- Extremely important
- Very important
- Somewhat important
- Not so important
- Not at all important

Comments/Suggestions

* 13. Station 7 lymphadenectomy: how difficult is the lymph node dissection along the left gastric artery and vein?

- Very easy
- Easy
- Neither easy nor difficult
- Difficult
- Very difficult

Comments/Suggestions

* 14. Station 7 lymphadenectomy: how important is the division of the left gastric vein at the upper border of the pancreas?

- Extremely important
- Very important
- Somewhat important
- Not so important
- Not at all important

Comments/Suggestions

* 15. Station 9 lymphadenectomy: how difficult is the lymph node dissection along the celiac trunk?

- Very easy
- Easy
- Neither easy nor difficult
- Difficult
- Very difficult

Comments/Suggestions

* 16. Stations 5 and 6 lymphadenectomies: how difficult is the lymph node dissection along the inferior and superior border of the proximal duodenum?

- Very easy
- Easy
- Neither easy nor difficult
- Difficult
- Very difficult

Comments/Suggestions

* 17. How difficult is it to perform a sufficient lymph node dissection along the common hepatic artery (station 8a)?

- Very easy
- Easy
- Neither easy nor difficult
- Difficult
- Very difficult

Comments/Suggestions

* 18. How difficult is it to perform a sufficient lymph node dissection along the proper hepatic artery (station 12a)?

- Very easy
- Easy
- Neither easy nor difficult
- Difficult
- Very difficult

Comments/Suggestions

* 19. How important is the dissection of the right (station 1) and left (station 2) paracardial lymph node dissection along the right and left crus?

- Extremly important
- Very important
- Somewhat important
- Not so important
- Not important at all

Comments/Suggestions

* 20. How important is the station 11d lymphadenectomy?

- Extremely important
- Very important
- Somewhat important
- Not so important
- Not at all important

Comments/Suggestions

* 21. Station 11d lymphadenectomy: how important is the division of the posterior gastric vessels at their origin from the splenic artery?

- Extremely important
- Very important
- Somewhat important
- Not so important
- Not at all important

Comments/Suggestions

* 22. How difficult is the lymph node dissection along the distal splenic artery (station 11d) without splenectomy?

- Very easy
- Easy
- Neither easy nor difficult
- Difficult
- Very difficult

Comments/Suggestions

* 23. How important is the lymphadenectomy of station 10 in patients with tumors of greater curvature or close to the spleen?

- Extremely important
- Very important
- Somewhat important
- Not so important
- Not at all important

Comments/Suggestions

* 24. Is the lymphadenectomy of station 10 important in patients with tumors that are not close to the greater curvature or spleen?

- Extremely important
- Very important
- Somewhat important
- Not so important
- Not at all important

Comments/Suggestions

* 25. How important is the omentectomy irrespective of the tumor stage?

- Extremely important
- Very important
- Somewhat important
- Not so important
- Not at all important

Comments/Suggestions

* 26. How important is the omentectomy for advanced tumor stages (T3/T4)?

- Extremely important
- Very important
- Somewhat important
- Not so important
- Not at all important

Comments/Suggestions

* 27. General aspects of the anastomosis: Should frozen section always be performed to ensure negative margins?

- Yes
- No

Comments/Suggestions

* 28. General aspects of the anastomosis: Should the frozen section be performed if the tumor location is perceived close to the margin?

- Yes
- No

Comments/Suggestions

* 29. General aspects of the anastomosis: Is a tumor-free resection margin sufficient after neoadjuvant treatment irrespective of the distance from margin?

- Yes
- No

Comments/Suggestions

* 30. General aspects of the anastomosis: Is a tumor free resection margin sufficient without neoadjuvant treatment irrespective of the distance from margin?

- Yes
- No

Comments/Suggestions

* 31. General aspects of anastomosis: How important is the assessment of the vascularization of the anastomosis with visual assessment?

- Extremely important
- Very important
- Somewhat important
- Not so important
- Not at all important

Comments/Suggestions

* 32. General aspects of anastomosis: How important is the assessment of the vascularization of the anastomosis with intraoperative imaging such as fluorescence or hyperspectral camera imaging?

- Extremely important
- Very important
- Somewhat important
- Not so important
- Not at all important

Comments/Suggestions

* 33. General aspects of the anastomosis: Do you perform a routine leakage test?

- Yes
- No

Comments/Suggestions

* 34. General aspects of the anastomosis: How important is the leakage test if performing the anastomosis was technically challenging?

- Extremely important
- Very important
- Somewhat important
- Not so important
- Not at all important

Comments/Suggestions
